# Ankyrin Repeat Domain 1 Overexpression is Associated with Common Resistance to Afatinib and Osimertinib in EGFR-mutant Lung Cancer

**DOI:** 10.1038/s41598-018-33190-8

**Published:** 2018-10-05

**Authors:** Akiko Takahashi, Masahiro Seike, Mika Chiba, Satoshi Takahashi, Shinji Nakamichi, Masaru Matsumoto, Susumu Takeuchi, Yuji Minegishi, Rintaro Noro, Shinobu Kunugi, Kaoru Kubota, Akihiko Gemma

**Affiliations:** 1 Division of Pulmonary Medicine and Oncology, Graduate School of Medicine, Nippon Medical School, Bunkyo-ku, Tokyo Japan; 2 Division of Pathology, Graduate School of Medicine, Nippon Medical School, Bunkyo-ku, Tokyo Japan

## Abstract

Overcoming acquired resistance to epidermal growth factor receptor tyrosine kinase inhibitors (EGFR-TKIs) is critical in combating EGFR-mutant non-small cell lung cancer (NSCLC). We tried to construct a novel therapeutic strategy to conquer the resistance to second-and third-generation EGFR-TKIs in EGFR-positive NSCLC patients. We established afatinib- and osimertinib-resistant lung adenocarcinoma cell lines. Exome sequencing, cDNA array and miRNA microarray were performed using the established cell lines to discover novel therapeutic targets associated with the resistance to second-and third-generation EGFR-TKIs. We found that ANKRD1 which is associated with the epithelial-mesenchymal transition (EMT) phenomenon and anti-apoptosis, was overexpressed in the second-and third-generation EGFR-TKIs-resistant cells at the mRNA and protein expression levels. When ANKRD1 was silenced in the EGFR-TKIs-resistant cell lines, afatinib and osimertinib could induce apoptosis of the cell lines. Imatinib could inhibit ANKRD1 expression, resulting in restoration of the sensitivity to afatinib and osimertinib of EGFR-TKI-resistant cells. In EGFR-mutant NSCLC patients, ANKRD1 was overexpressed in the tumor after the failure of EGFR-TKI therapy, especially after long-duration EGFR-TKI treatments. ANKRD1 overexpression which was associated with EMT features and anti-apoptosis, was commonly involved in resistance to second-and third-generation EGFR-TKIs. ANKRD1 inhibition could be a promising therapeutic strategy in EGFR-mutant NSCLC patients.

## Introduction

Lung cancer is the leading cause of death in Japan^1^. Up to 69% of the patients with advanced non-small-cell lung cancer (NSCLC) could have a potentially actionable molecular target^2,3^. The most frequent EGFR-sensitizing mutations are exon 19 deletions and exon 21 L858R mutation^4,5^. Treatment with EGFR-receptor tyrosine kinase inhibitors (EGFR-TKIs), such as first- or second-generation EGFR-TKIs, gefitinib and erlotinib^6–9^ or afatinib^10,11^ respectively, led to a dramatic clinical response and improvement of prognosis in EGFR-mutant NSCLC patients. However, most patients treated with first-or second-generation EGFR-TKIs showed disease progression within one year due to acquired drug resistance. The gatekeeper Thr790Met mutation (T790M) is responsible for almost 60% of the resistant mechanisms against EGFR-TKIs^12^. The third-generation EGFR-TKI, osimertinib, targets T790M and EGFR-activating mutations. The AURA3 phase III trial showed that progression free survival (PFS) was significantly longer in patients treated with osimertinib (median,10.1 months) than in patients treated with platinum therapy plus pemetrexed (4.4 months)^13^. The C797S secondary mutation was already reported to be 20–30% of the mechanism of resistance to osimertinib in T790M-positive NSCLC patients. However, the mechanism of the resistance to osimertinib has not been clarified yet^14^. Therefore, a therapeutic strategy to overcome the origins of resistance is critical for the elimination of EGFR-mutant NSCLC.

In the present study, we aimed to clarify the common mechanism of the development of acquired resistance to second-and third-generation EGFR-TKIs and to demonstrate potential therapeutic strategies to conquer EGFR-mutant NSCLCs. We established afatinib- and osimertinib-resistant adenocarcinoma cell lines showing overexpression of ankyrin repeat domain 1 (ANKRD1), which was associated with epithelial-mesenchymal transition (EMT) and anti-apoptosis. Inhibition of ANKRD1 led to induction of apoptosis by afatinib or osimertinib in adenocarcinoma cells, providing a critical therapeutic target for resistance to EGFR-TKIs in lung adenocarcinoma.

## Results

### Effect of afatinib and osimertinib on lung adenocarcinoma cell lines and establishment of resistant cell lines

**Figure 1:**
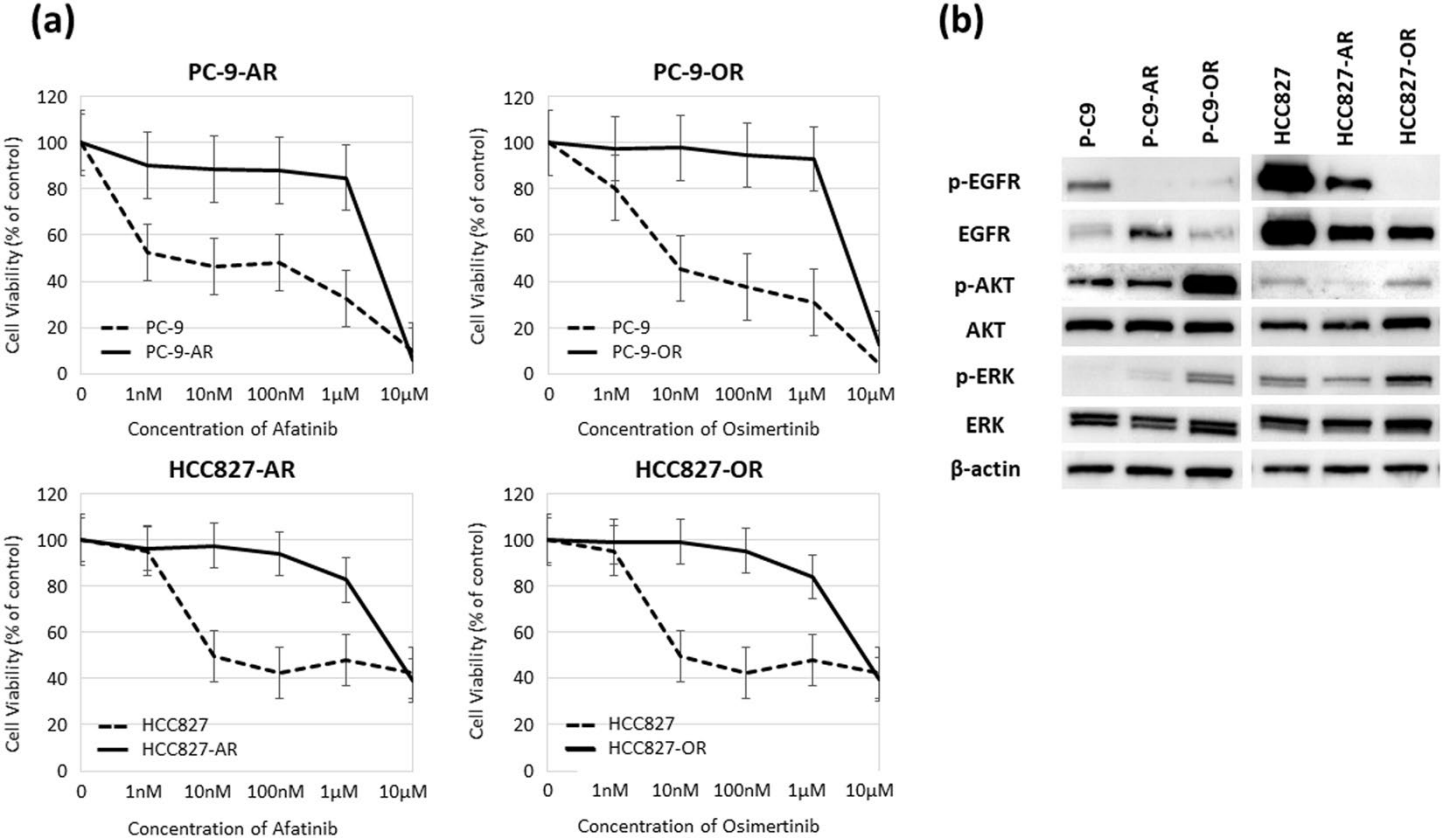
Establishment of afatinib- and osimertinib-resistant PC-9 and HCC-827 cells. (**a**) PC-9-afatinib-resistant cell line (PC-9-AR); PC-9-osimertinib-resistant cell line (PC-9-OR); HCC827-afatinib-resistant cell line (HCC827-AR); and HCC827-osimertinib-resistant cell line (HCC827-OR). The results of cell viability assays are shown. (**b**) Protein expression levels of EGFR signal pathway molecules.

We evaluated the antitumor activities of afatinib and osimertinib in two EGFR-mutant lung adenocarcinoma cell lines, PC-9 and HCC827, by MTS assay. Based on the IC_50_ values, we tried to establish afatinib- or osimertinib-resistant cell lines using a stepwise method^15,16^. Parental cell lines were revealed by low dose medicine (10% density of IC50), and they were raised quantity of revelation by degrees. After 6 months, four drug-resistant cell lines were established: PC-9-afatinib (PC-9-AR), PC-9-osimertinib (PC-9-OR), HCC827-afatinib (HCC827-AR) and HCC827-osimertinib (HCC827-OR) resistant cell lines (Supplemental Fig. S1). We confirmed the resistant cell lines were stable at least 6-month period without adding any substance. Figure 1a shows a comparison of the dose-dependent sensitivity to each drug in the original cell lines and established resistant cell lines. Table 1 shows the IC_50_ values of the parental and resistant cell lines. Afatinib and osimertinib showed anti-tumor activities in the parental cell lines as the IC50 values were <10 nM. In contrast, 400- to 900-fold resistance to afatinib or osimertinib was revealed in the established drug-resistant cell lines. No secondary mutations including T790M and C797S were found by whole exome sequencing. Furthermore, EGFR minor mutations such as EGFR L718Q and G796D and some novel mutations including MET amplification and BRAF V600E were not also observed in four resistant NSCLC cells. Figure 1b shows protein expression levels of EGFR signal pathway molecules in the parental cells and EGFR-TKIs-resistant cells. The protein level of p-EGFR was diminished in the afatinib and osimertinib-resistant cell lines, and the protein levels of p-AKT and p-ERK were increased in the two osimertinib-resistant cell lines. These findings demonstrated that activation of another bypassing pathway, but not secondary mutation including T790M and C797S, was mainly involved in the drug resistance to afatinib and osimertinib of EGFR-mutant NSCLC cells.

**Table 1:** IC_50_ values and T790M/C797S status in EGFR-TKIs-resistant cell lines.

	IC_50_ value (μM) (Mean ± SD)	*p*-value	EGFR mutation
PC-9	0.0033 ± 0.0016	<0.001	a deletion in exon 19
PC-9-AR	2.7 ± 0.34		a deletion in exon 19
Fold change	818		
PC-9	0.0072 ± 0.0011	<0.001	a deletion in exon 19
PC-9-OR	3.5 ± 0.37		a deletion in exon 19
Fold change	486		
HCC827	0.01 ± 0.0013	<0.001	a deletion in exon 19
HCC827-AR	5.7 ± 1.2		a deletion in exon 19
Fold change	570		
HCC827	0.01 ± 0.0013	<0.001	a deletion in exon 19
HCC827-OR	6.1 ± 2.0		a deletion in exon 19
Fold change	610		

### Overexpression of ANKRD1 in EGFR-TKIs-resistant cell lines

**Figure 2:**
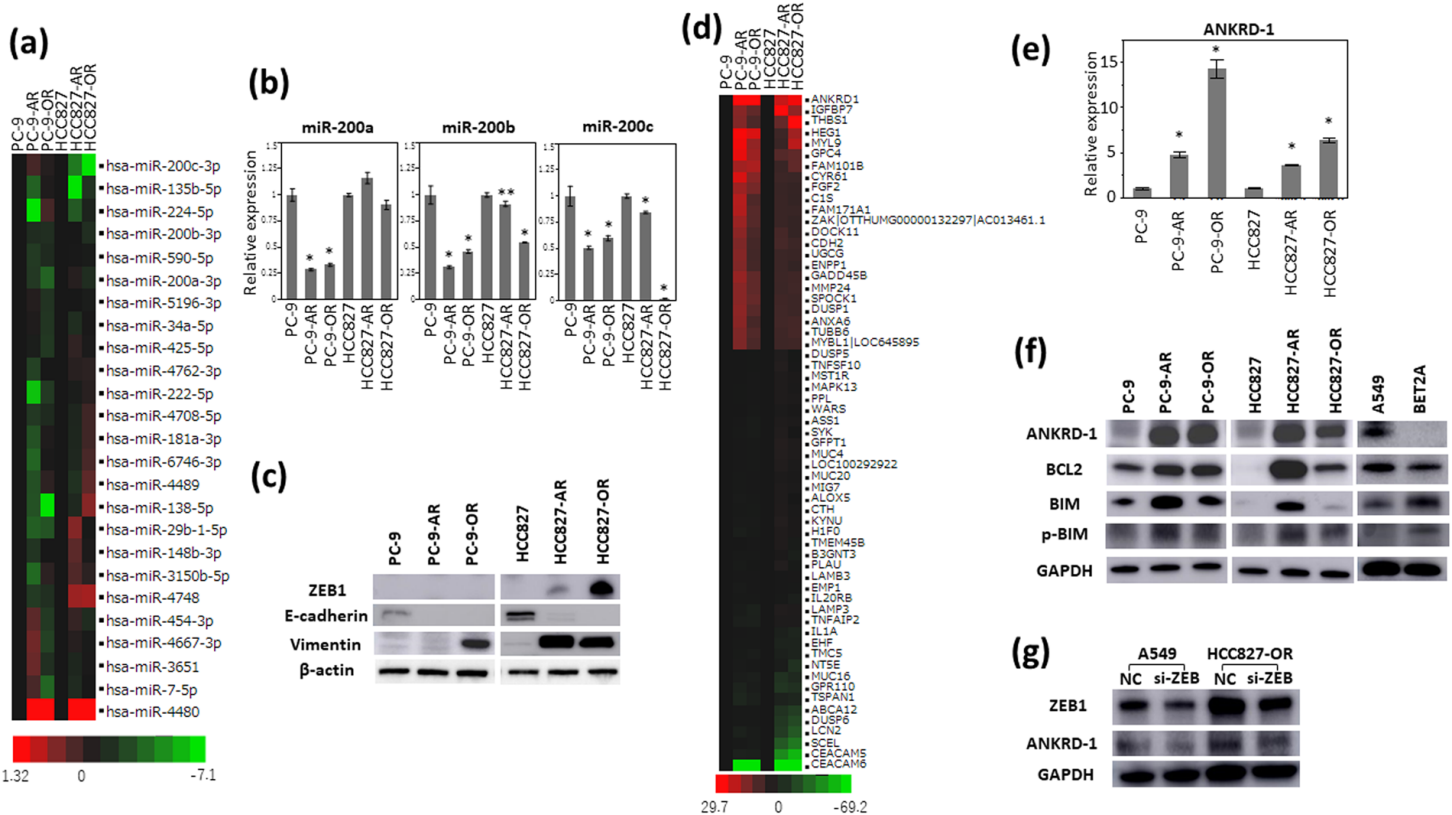
ANKRD1 overexpression the four EGFR-TKIs-resistant cell lines. (**a**) The miRNA microarray analysis showed that expression of the miR-200 family were downregulated in EGFR-TKIs-resistant cell lines. (**b**) The expression of miR-200a, miR-200b and miR-200c by qRT-PCR analysis. *p < 0.01, **p < 0.05. (**c**) Protein expression of factors related to epithelial-mesenchymal transition. (**d**) cDNA microarrays showed gene expression profiles in the parental and four resistant cell lines. (**e**) qRT-PCR analysis showed overexpression of ANKRD1 in the resistant cell lines. *p < 0.01. (**f**) Upregulation of ANKRD1 proteins was shown by Western blotting analysis. (**g**) ZEB1 expression in A549 and HCC827-OR before and after siZEB1 transfection for 72 hours. The results of Western blotting analysis are shown. NC: siRNA negative control.

We first performed miRNA microarray analysis to evaluate the post-transcriptional regulation associated with drug resistance using the parental and EGFR-TKIs-resistant NSCLC cells. miR-200a and miR-200b were remarkably downregulated in the osimertinib-resistant cell lines (Fig. 2a). Decreased expression of miR-200a, miR-200b and miR-200c were observed in the osimertinib-resistant cell lines by qRT-PCR (Fig. 2b). The miR-200 family has been reported to be associated with epithelial-mesenchymal transition (EMT) by targeting ZEB1^16^. We examined the expression of EMT markers including ZEB1, E-cadherin and vimentin by Western blotting analysis. E-cadherin was diminished in the afatinib- and osimertinib-resistant cells. ZEB1 was increased in HCC827-AR and HCC827-OR. Increased expression of vimentin was observed in PC9-OR, HCC827-AR and HCC827-OR, suggesting the presence of EMT phenomenon in EGFR-TKIs-resistant cells (Fig. 2c).

We next performed cDNA microarray analysis to identify the genes associated with resistance to afatinib and osimertinib using the parental and EGFR-TKIs-resistant NSCLC cells. ANKRD1 was the most and commonly upregulated gene in the four EGFR-TKIs-resistant cell lines (Fig. 2d). ANKRD1 was significantly overexpressed in the four EGFR-TKIs-resistant cell lines on qRT-PCR analysis (Fig. 2e). ANKRD1 protein expression was also upregulated in the four EGFR-TKIs-resistant cell lines compared with the parental cell lines (Fig. 2f). To confirm the robustness of upregulated ANKRD1 in EGFR-TKIs-resistant cells, cloned EGFR-TKI-resistant cells derived from a single cell by limiting dilution method were generated. Three independent clones of each EGFR-TKI-resistant cell lines also showed increased protein levels of ANKRD1 by western blotting analysis (Supplemental Fig. S2). We evaluated migration of resistant cells by CytoSelect^TM^ 24-Well Cell Haptotaxis Assay (Cell Biolabs, INC.; CA). HCC827-AR and HCC827-OR were increased migration abilities than parental cell lines, PC-9-AR and PC9-OR had the tendency to increase migration abilities (Supplemental Fig. S3). Correlation between the expression levels of ANKRD1 and EMT marker ZEB1 was previously reported^17^. After ZEB1 silencing by the specific siRNA, the ANKRD1 protein level was decreased as well as ZEB1 suppression (Fig. 2g, Supplemental Fig. S4). ANKRD1 has activity to in anti-apoptosis^18–20^. The level of BCL-2 protein which is related to down regulation of apoptosis, was higher in the four EGFR-TKIs-resistant cell lines than in the parental cell lines (Fig. 2f). BIM protein is known as an apoptotic instruction factor. The level of p-BIM which offsets induction of apoptosis^21,22^, was also more greatly increased in the resistant cell lines than in the parental cell lines (Fig. 2f). The A549 EGFR wild type cell line showed similar protein levels of ANKRD1, BCL2, BIM and p-BIM as those in the resistant cell lines (Fig. 2f). The BET2A (normal lung cell lines) showed low protein level of ANKRD1 (Fig. 2f). These results suggested that ANKRD1 overexpression is associated with the EMT phenomenon, and anti-apoptosis was mainly involved in the resistance to afatinib and osimertinib of EGFR-mutant NSCLC cells.

### Knockdown of ANKRD1 led to induction of apoptosis by EGFR-TKIs and overcame the resistance to EGFR-TKIs

**Figure 3:**
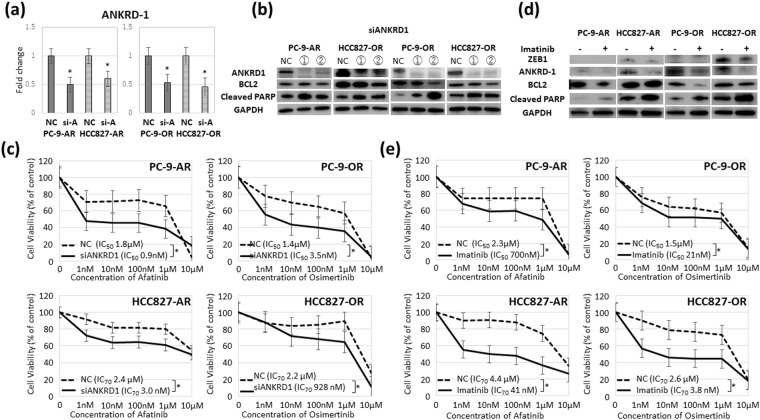
Suppression of ANKRD1 overcame the resistance to EGFR-TKIs. (**a**) ANKRD1 levels in PC9-AR, HCC827-AR, PC9-OR and HCC827-OR before and after siANKRD1-2 transfection by qRT-PCR. *p < 0.01. (**b**) Western blot analysis of BCL-2 and cleaved PARP after ANKRD1 silencing. ANKRD1 was silenced after siANKRD1 transfection for 96 hours. (**c**) The dose-dependent sensitivity to afatinib and osimertinib in the resistant cell lines after ANKRD1 siRNA treatment. *p < 0.01. (**d**) **Afatinib and o**simertinib-resistant cell lines were treated with **afatinib (500 nM) or** osimertinib (500 nM) with or without imatinib (1 μM) for 24 hours. (**e**) The dose-dependent sensitivity to afatinib and osimertinib in the resistant cell lines with or without imatinib. NC: siRNA negative control, si-A: siANKRD1-2 *p < 0.01.

We next evaluated whether ANKRD1 inhibition led to recovery of apoptosis and sensitivity to EGFR-TKIs. Figure 3a shows the suppression of ANKRD1 by siANKRD1 in PC-9-AR, PC-9-OR, HCC827-AR and HCC827-OR. After ANKRD1 silencing, decreased level of BCL-2 and increased level of cleaved PARP indicating apoptotic activity were observed in the four resistant cell lines (Fig. 3b). Knockdown of ANKRD1 did not change the expression of E-cadherin and vimentin (data not shown). Figure 3c shows a comparison of the dose-dependent sensitivity of the EGFR-TKIs-resistant cell lines to afatinib and osimertinib after ANKRD1 inhibition by siRNA. Silencing of ANKRD1 could overcome the resistance to afatinib and osimertinib of EGFR-TKI-resistant cell lines, except for HCC-827-OR.

We also searched clinically available compounds that can overcome the resistance to afatinib or osimertinib using the SCADS inhibitor kit III (Table 2). Platelet-derived growth factor (PDGFR) tyrosine kinase inhibitor IV suppressed growth of the resistant cell line. Imatinib is a multi-target inhibitor of tyrosine kinase targeting PDGFR, v-Abl, and c-Kit^23–25^. Imatinib could inhibit ZEB1 and ANKRD1 expression (Fig. 3d). Increased levels of BCL-2 and cleaved PARP indicating apoptotic activity were found after the imatinib treatment in four resistant cell lines (Fig. 3d). Imatinib could also restore the sensitivity to afatinib and osimertinib in EGFR-TKI-resistant cell lines (Fig. 3e).

**Table 2:** Screening analysis using 95chemical compounds at 500 nM to clarify the candidate molecules for EGFR-TKIs-resistant cells with the SCADS Inhibitor Kit III.

Target	Compound	Relative expression			
		PC-9-AR	PC-9-OR	HCC827-AR	HCC827-OR
PDGFR	PDGFR tyrosine kinase inhibitor IV	0.14	0.14	0.74	0.73
AKT	AKT inhibitor IV	0.38	0.44	1.15	1.25
CDK	Cdk 1/2 inhibitor III	0.38	0.27	0.70	1.64
Chk	SB218078	0.77	0.86	0.65	1.08
EGFR	AG1478	2.60	1.69	1.12	2.10
Hsp90	Radicicol	1.39	0.98	0.99	1.59

### Frequency of ANKRD1-positive cancer cells in surgically or bronchoscopically obtained specimens from EGFR-mutant NSCLC patients

**Figure 4:**
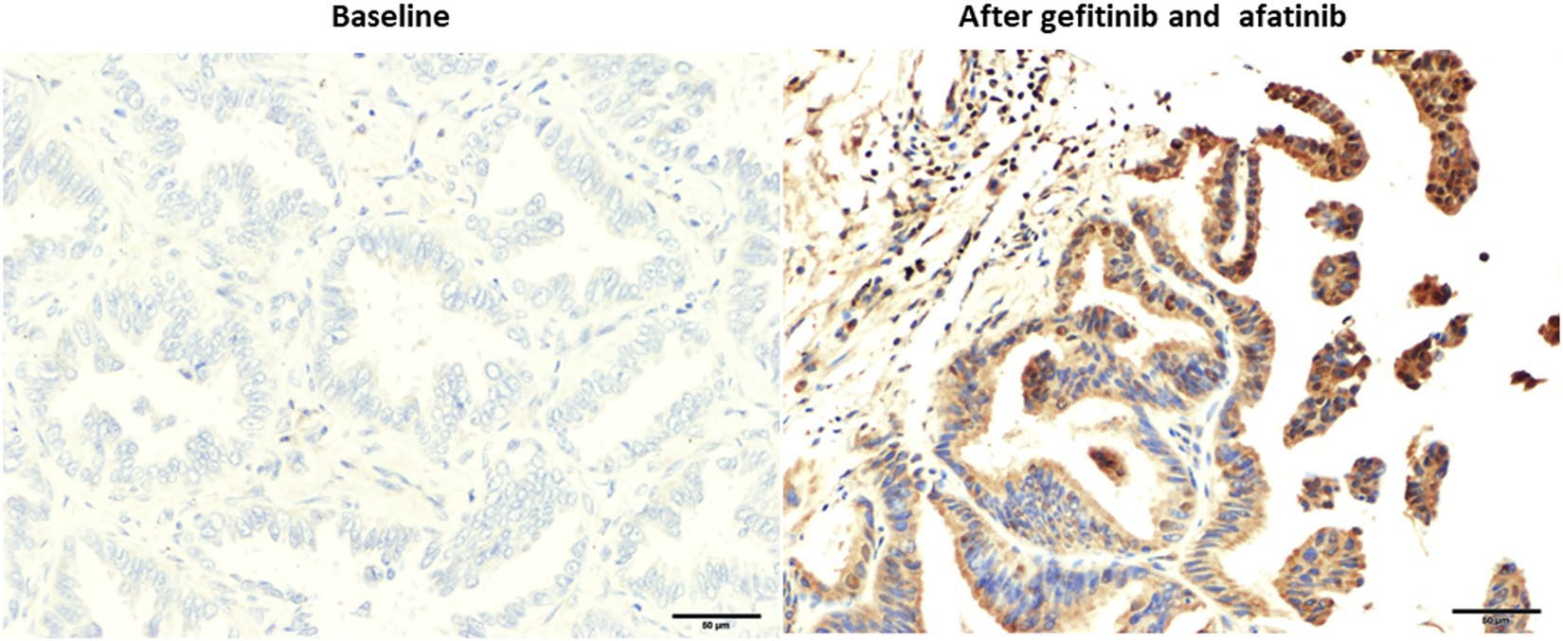
IHC staining for ANKRD1 in lung cancer specimens obtained from patient No.9. Negative ANKRD1 staining before EGFR-TKIs treatment (left), and positive ANKRD1 staining after gefitinib and afatinib therapy (right).

We finally investigated the ANKRD1 expression status in EGFR-mutant NSCLC patients before and after the EGFR-TKI therapy by IHC analysis (Table 3). We confirmed positive-ANKRD1 expression using commercially available myocardium tissue (Supplemental Fig. S5). Figure 4 shows the ANKRD1 expression status of patient No. 9 after gefitinib and afatinib treatments over 52 months. Significantly increased ANKRD1 expression was observed after EGFR-TKIs therapy compared with the baseline expression level. Eight of 10 patients who were treated with EGFR-TKIs for more than 6 months showed increased expression of ANKRD1 after the treatment (No.3-No.10) (Table 3). Four of these 8 patients showed also increased expression of vimentin after the treatment (No.3, 4, 6, 10) (Supplemental Table 1). On the other hand, the level of ANKRD1 expression was not changed in 2 patients (No.1 and No.2) who were treated with EGFR-TKIs for less than 6 months (Table 3). Baseline high levels of ANKRD1 and low levels of E-cadherin suggesting in EMT phenotype may reflect on the intrinsic resistance to EGFR-TKIs in these 2 patients (Supplemental Fig. 6 and Table 1). Although the sample size was small, these results suggested that long-term EGFR-TKI treatment might induce ANKRD1 expression which is involved in the drug resistance.

**Table 3:** ANKRD1 expression levels in lung specimens changed after EGFR-TKI therapy.

No.	Gender	EGFR mutation	EGFR-TKIs Therapy		Score of Immunostaining (%)		
					Baseline	After EGFR-TKI	Fold change
1	Male	L858R	Gefitinib	3 months	78	75	0.96
2	Female	L858R	Afatinib	5 months	70	84	1.2
3	Male	Del19	Gefitinib Erlotinib Afatinib	5 months 4 months 4 months	36	84	2.3
4	Female	Del19	Gefitinib	22 months	1	98	98
5	Female	Del19	Gefitinib	22 months	5	88	17.6
6	Female	Del19	Gefitinib Erlotinib	10 months 24 months	1	100	100
7	Male	Del19	Gefitinib Erlotinib Afatinib	34 months 4 months 3 months	0	88	>100
8	Female	Del19	Erlotinib	50 months	39	78	2.0
9	Male	Del19	Gefitinib Afatinib	48 months 4 months	13	100	7.7
10	Female	Del19	Gefitinib Afatinib	58 months 12 months	25	62	2.5

## Discussion

First-or second-generation EGFR-TKIs are key drugs for NSCLC patients with EGFR mutation^6–11^. However, overcoming the drug resistance has remained as a major issue. Osimertinib which was originally developed for tumors that developed tolerance to first- or second-generation EGFR-TKIs, targeting T790M, showed a dramatic response in T790M-positive NSCLC patients^13,26^. However, the C797S mutation was already observed as a mechanism of resistance in T790M-positive NSCLC patients after osimertinib treatment^14^. A recent report demonstrated that brigatinib combined with anti-EGFR antibody could rescue osimertinib resistance by overcoming all of the activating-mutations, T790M and C797S, in EGFR-mutated NSCLC patients^27^. However, C797S was found in only 20% in osimertinib-resistant NSCLC patients. The battle between drug-resistant additional mutations and molecular-targeted agents is not getting anywhere. Therefore, we wanted to develop a fundamental strategy to overcome the origins of drug resistance in EGFR-mutant NSCLC.

Recently, a drug-tolerant cancer cell subpopulation commonly affected by all EGFR-TKIs was recognized as a critical target. A recent study demonstrated that EGFR T790M mutation as a resistant mechanism can occur in both pre-existing T790M clones and in the evolution of drug-tolerant cells without T790M^28^. Late-evolving EGFR T790M in cancer cells that acquired resistance showed a decreased apoptotic response to EGFR-TKI. Therefore, overcoming resistance of the drug-tolerant cell subpopulation is critical for elimination of EGFR-mutant lung cancer.

In this study, we established afatinib- or osimertinib-resistant cell lines from EGFR-mutant PC-9 and HCC827 cells. In these resistant cells, no secondary gatekeeper mutations such as T790M and C797S were observed. We ultimately found that ANKRD1 upregulation, which was associated with EMT and anti-apoptosis, was critical to the resistance against afatinib and osimertinib in EGFR-mutant cells. Osimertinib induced EMT phenomenon has been reported^29^. ANKRD1, also known as cardiac ankyrin repeat protein or cardiac adriamycin responsive protein, is a protein encoded by the ANKRD1 gene (10q23.31) in humans. It is mainly expressed in cardiac and skeletal muscles and its expression is increased in human heart failure such as idiopathic dilated cardiomyopathy^30,31^. As for cancer progression, ANKRD1 was shown to be a co-activator of the *p53* tumor suppressor protein^32^. ANKRD1 overexpression was also associated with resistance to cisplatin and worse outcome of ovarian cancer^33^. Furthermore, ANKRD1 plays a significant role in regulation of apoptosis in human ovarian cancer cells^34^. In the present study, we first demonstrated that ANKRD1 was commonly involved in anti-apoptosis and resistance to EGFR-TKIs in lung adenocarcinoma. Overexpression of ANKRD1 was noted in the specimens from the EGFR-mutant patients after the failure of EGFR-TKI therapy, especially after long-duration EGFR-TKI treatments. ANKRD1 expression could be induced by the stress response^35^. Long-term exposure of lung cancer cells to EGFR-TKIs may lead to accumulation of ANKRD1 protein. ANKRD1 inhibition or imatinib targeting ANKRD1 could rescue the acquired resistance to afatinib or osimertinib in EGFR-mutant cells.

Imatinib can be used as the treatment for chronic myelogenous leukemia or gastrointestinal stromal tumors in the clinical setting. Imatinib was reported to inhibit PDGFR and ZEB1^36,37^. PDGFRα could activate ZEB1 expression^38^. Correlation between the expression of ANKRD1 and ZEB1 was reported in breast cancer^17^. Imatinib treatment led to downregulation of ZEB1 and ANKRD1 in two EGFR-TKIs-resistant cell lines. Suppression of anti-apoptotic Bcl-2 and the appearance of cleavage PARP were observed after treatment with imatinib. Recovery of the sensitivity to afatinib and osimertinib was also observed after imatinib treatment. These results could provide a new insight into the possibility of therapy with imatinib combined with EGFR-TKIs, which might lead EGFR-mutated NSCLC patients to a cure by overcoming the origin of resistance.

Our study had a limitation. ANKRD1 expression status before and after the EGFR-TKI therapy including gefitinib and afatinib, but not osimertinib, have been evaluated using only 10 NSCLC patients. A further study will be performed to confirm this significance of ANKRD1 on the resistance to osimertinib using large scale EGFR-positive NSCLC patients.

In conclusion, we found that ANKRD1 overexpression which was associated with EMT features and anti-apoptosis, was commonly involved in resistance to the second-and third-generation EGFR-TKIs, afatinib and osimertinib, respectively. ANKRD1 overexpression also contributed to acquired resistance to the second-and third-generation EGFR-TKIs in EGFR-positive NSCLC patients. Imatinib could inhibit ANKRD1 expression, resulting in the induction of apoptosis and overcoming drug resistance to EGFR-TKIs. ANKRD1 might be closely correlated with the drug-tolerant subpopulation of cancer cells which is commonly involved in the drug resistance to EGFR-TKIs. Therefore, ANKRD1 inhibition could be a promising new therapeutic strategy to conquer EGFR-mutant NSCLCs.

## Materials and Methods

### Cell cultures

Three human lung adenocarcinoma cell lines were used in the present study. PC-9 with an exon 19 in-frame deletion was provided from Immuno-Biological Laboratories (Gunma, Japan). NCI-HCC827 (HCC827) with a deletion in exon 19, A549 (EGFR wild type cell line) and BET2A (normal lung cell line) were purchased from the American Type Culture Collection (ATCC, Manassas, VA). PC-9 and HCC827 were cultured in RPMI1640 (Wako, Osaka, Japan) containing 10% fetal bovine serum (FBS; BioWest, Nuaille, France) and 1% penicillin and streptomycin (Wako Pure Chemical Industries, Osaka, Japan). These cell lines were obtained from 2010 through 2016, amplified and frozen, and one aliquot of each was thawed for this research. All cells were routinely screened for the absence of mycoplasma, and at least three independent experiments were performed for each condition.

### Drugs and growth-inhibition assay

Afatinib, osimertinib and imatinib were purchased from Selleck Chemicals (Houston, TX). The MTS assay was used to assess the growth inhibition effects of afatinib and osimertinib on the two adenocarcinoma cell lines as previously described^15,39^. Cell suspensions (5,000 cells/well) were seeded into 96-well plates and drugs of increasing concentrations (0, 0.001, 0.01, 0.1, 1 and 10 μM) were added. The half maximal inhibitory concentration (IC_50_) value was defined as the concentration of afatinib or osimertinib needed for 50% reduction of growth. The corrected absorbance of each sample compared with that of the untreated control was calculated. Each experiment was performed independently three times.

Screening analysis was done using 95 chemical compounds at 500 nM in the resistant cell lines with the SCADs inhibitor kit III (Screening Committee of Anticancer Drug; Tokyo, Japan) as previously described^40^.

### Exome sequencing

DNA was extracted from cultured cell lines using a QIAamp DNA mini kit (QIAGEN, Hilden, Germany) according to the manufacturer’s protocol. Exome sequencing of extracted DNA was conducted on an Illumina HiSeq. 2500 platform using paired-end reads (Illumina, San Diego, CA). Reads were aligned against the reference human genome and compared with each cell line.

### Western blotting analysis

Protein extraction, 2D-PAGE and transfer to nitrocellulose membranes were performed as previously described^15,39^. The membrane was incubated with the following antibodies. Antibodies against EGFR, phosphorylated EGFR (p-EGFR), AKT, p-AKT, ERK, p-ERK, ZEB-1, E-cadherin, vimentin, BIM, p-BIM, and cleaved PARP were purchased from Cell Signaling Technology (Danvers, MA). Antibodies against ANKRD1, GAPDH and BCL2 were obtained from Santa Cruz Biotechnology (Santa Cruz, CA), and antibodies against β-actin from Sigma-Aldrich (St. Louis, MO).

### RNA extraction and Real-time quantitative reverse transcription PCR

RNA was extracted from the adenocarcinoma cell lines by TRIzol reagent (Thermo Fisher Scientific, Waltham, MA) as previously described^41^. The cDNAs were used for quantitative reverse transcription PCR (qRT-PCR) analysis of ANKRD1 using THUNDERBIRD SYBR qPCR/RT Set III (TOYOBO, Osaka, Japan) as per the instruction manual. The expression levels of the miR-200 family were measured by TaqMan MicroRNA assay system (Thermo Fisher Scientific) as previously described^42^. Gene and miRNA expression were quantified as 2^−ΔΔ*C*^_t_ value.

### miRNA microarray and cDNA array analysis

miRNA analysis was performed using 3D-Gene Human miRNA oligo chips ver.21 (TORAY, Tokyo, Japan). cDNA array was approached based on the Affymetrix GeneChip (Thermo Fisher Scientific). Scanning RNA (total 250 ng) was performed with the 3D-Gene miRNA Labeling kit (TORAY) and 3D-Gene Scanner 3000. Microarray data have been deposited in NCBI’s Gene Expression Omnibus (GEO; http://www.ncbi.nlm.nih.gov/geo/) and are accessible through the GEO series accession number GSE 106765.

### Small-interfering RNA (siRNA) transfection

siRNA targeting ANKRD1, ZEB1 and negative control were synthesized by Thermo Fisher Scientific. All siRNA was treated with Lipofectamine 2000 (Thermo Fisher Scientific) transfection reagent 24 hours after seeding, as per the manufacturer’s instructions. The siRNA complexes were transfected into cells at a final concentration of 40 nM. The transfection medium was replaced 6 hours later, and cells were then incubated at 37 °C for 48 hours.

### Immunohistochemical analysis using surgically or bronchoscopically obtained specimens

During the period from 2009 through 2016, 10 lung adenocarcinoma patients with EGFR mutation underwent re-biopsy after EGFR-TKIs therapy at Nippon Medical School Hospital. Myocardium tissue, which was ANKRD1 positive control, was provided from Super Bio Chips (Seoul, Korea). ANKRD1 E-cadherin, and Vimentin expression was evaluated using tumor specimens obtained before and after EGFR-TKIs therapy by immunohistochemical analysis (IHC). IHC was performed as previously described^43^. The sections were stained overnight at 4 °C with rabbit anti-human ANKRD1 antibody (Aviva Systems Biology, San Diego, CA) at a final dilution of 1:200, E-cadeherin (Santa Cruz) at a final dilution of 1:50 and Vimentin (Cell Signaling Technology) at a final dilution of 1:50. Upon washing with PBS, the slides were incubated with biotinylated rabbit anti-mouse immunoglobulin IgG (dilution 1:200, Vector Laboratories Inc., Burlingame, CA) at room temperature for 30 minutes. The three most ANKRD1-positive areas within each section were selected, and the number of ANKRD1-positive tumor cells were counted under a light microscope at ×400 magnification (0.0625 mm^2^/field). Two observers (A. T. and R. N.) who were unaware of the clinical data independently reviewed the histologic specimens as previously described^44,45^. The study protocol was approved by the ethics committee review board at Nippon Medical School Hospital. Written informed consent was obtained from all the patients, and patient specimens were collected in accordance with the Declaration of Helsinki 2013.

### Statistical analysis

Differences in categorical outcomes were evaluated using the chi-square test. The standard Student *t*-test was used to determine the significance of differences compared with the control group. All *P* values were 2-sided, and the significance level was set at less than 0.05. Analyses were performed using the statistical software JMP 9 (SAS Institute, Cary, NC).

## Electronic supplementary material

Supplemental Figure
