# Supplementary material for: Ankyrin Repeat Domain 1 Overexpression is Associated with Common Resistance to Afatinib and Osimertinib in EGFR-mutant Lung Cancer
Source: Sci Rep. 2018 Oct 5;8:14896. doi: 10.1038/s41598-018-33190-8 (PMC6173712; doi:10.1038/s41598-018-33190-8)
Supplement: Supplementary file 1 — Supplemental Figure [file 41598_2018_33190_MOESM1_ESM.docx]

**Ankyrin Repeat Domain 1 Overexpression is Associated with Common Resistance to Afatinib and Osimertinib in EGFR-mutant Lung Cancer**

**Authors and affiliations:**

Akiko Takahashi^1^, Masahiro Seike^1^, Mika Chiba^1^, Satoshi Takahashi^1^, Shinji Nakamichi^1^,

Masaru Matsumoto^1^, Susumu Takeuchi^1^, Yuji Minegishi^1^, Rintaro Noro^1^, Shinobu Kunugi^2^,

Kaoru Kubota^1^, Akihiko Gemma^1^

^1^ Division of Pulmonary Medicine and Oncology, Graduate School of Medicine,

Nippon Medical School, Bunkyo-ku, Tokyo, Japan

^2^ Division of Pathology, Graduate School of Medicine,

Nippon Medical School, Bunkyo-ku, Tokyo, Japan

Akiko Takahashi: [s9056@nms.ac.jp](mailto:s9056@nms.ac.jp)

Masahiro Seike: [mseike@nms.ac.jp](mailto:mseike@nms.ac.jp)

Mika Chiba: [m-chiba0@nms.ac.jp](mailto:m-chiba0@nms.ac.jp)

Satoshi Takahashi: [satoshi506@nms.ac.jp](mailto:satoshi506@nms.ac.jp)

Shinji Nakamichi: [snakamichi@nms.ac.jp](mailto:snakamichi@nms.ac.jp)

Masaru Matsumoto: [s7062@nms.ac.jp](mailto:s7062@nms.ac.jp)

Susumu Takeuchi: [s-takeuchi@nms.ac.jp](mailto:s-takeuchi@nms.ac.jp)

Yuji Minegishi: [uminegis@nms.ac.jp](mailto:uminegis@nms.ac.jp)

Rintaro Noro: [r-noro@nms.ac.jp](mailto:r-noro@nms.ac.jp)

Shinobu Kunugi: [s-hmmi@nms.ac.jp](mailto:s-hmmi@nms.ac.jp)

Kaoru Kubota: [kkubota@nms.ac.jp](mailto:kkubota@nms.ac.jp)

Akihiko Gemma: agemma@nms.ac.jp

**Address correspondence to:** Masahiro Seike, MD, PhD

Division of Pulmonary Medicine and Oncology, Graduate School of Medicine,

Nippon Medical School, Bunkyo-ku, Tokyo, 113-8063, Japan

E-mail address: mseike@nms.ac.jp

**Supplemental Figure S1. EMT features in afatinib- and osimertinib-resistant cells.**

Morphologic changes are observed under a light microscope in parental and PC-9-AR, PC-9-OR, HCC827-AR and HCC827-OR cells.


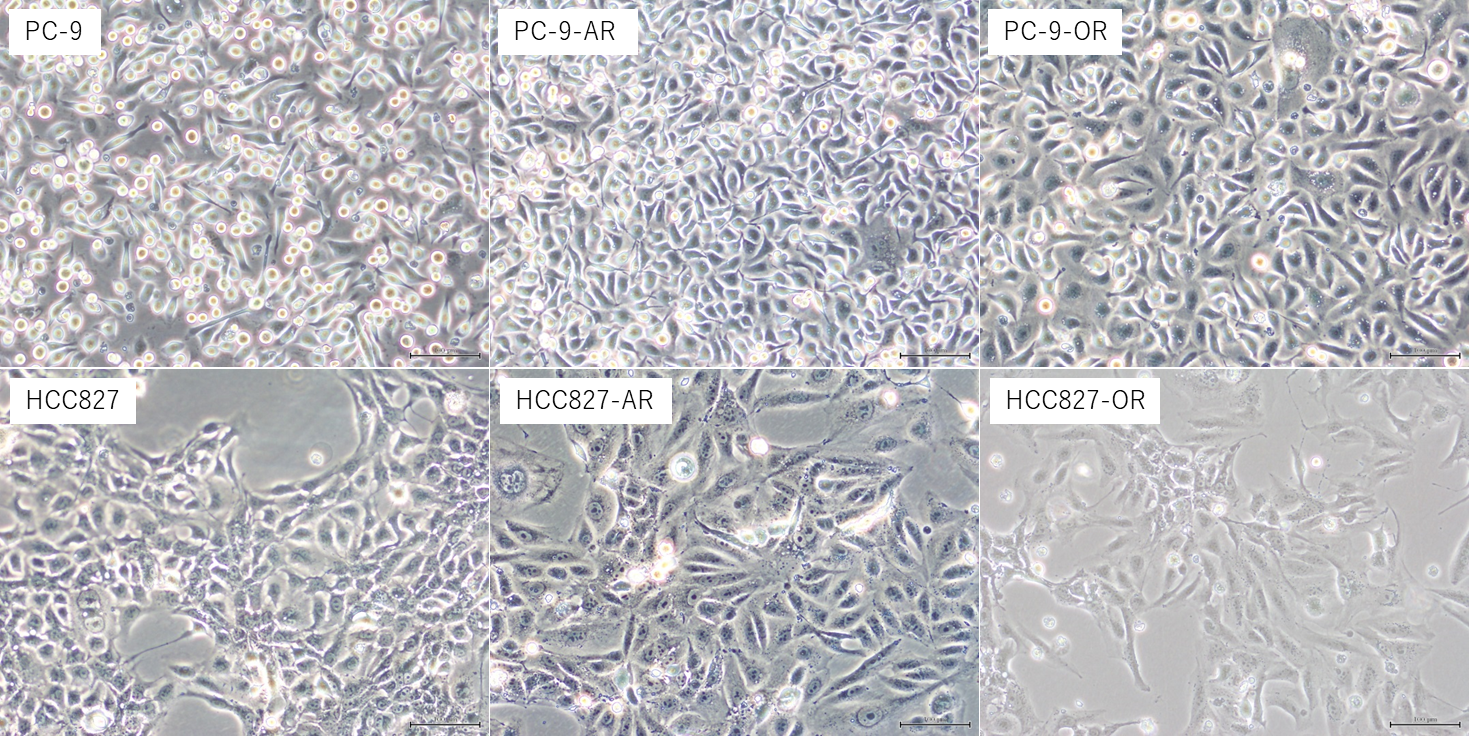


**Supplemental Figure S2. Upregulated ANKRD-1 protein was confirmed in cloned afatinib- and osimertinib-resistant cells.**

Western blot analysis of ANKRD1 protein in PC-9-AR, PC-9-OR, HCC827-AR and HCC827-OR cells were shown. Original: original resistant cells. # No.: cloned resistant cells.


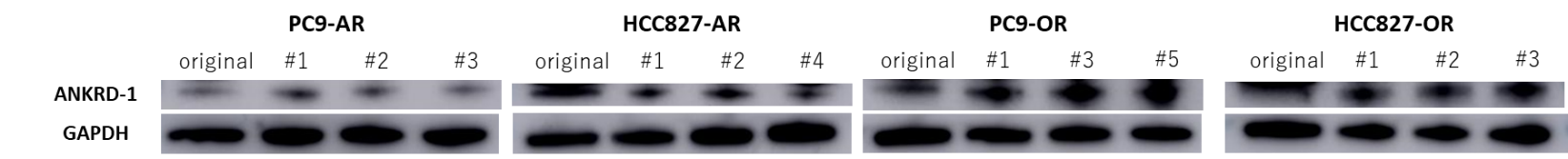


**Supplemental Figure S3. Increased migration abilities in afatinib- and osimertinib-resistant cells.**

Relative rations of migration abilities of afatinib- and osimertinib-resistant PC-9 and HCC-827 cells in comparison with parental cells at absorbance 560nm were shown. HCC827-AR and HCC827-OR were significant increased migration abilities than parental cell lines. * *p* < 0.01.

**
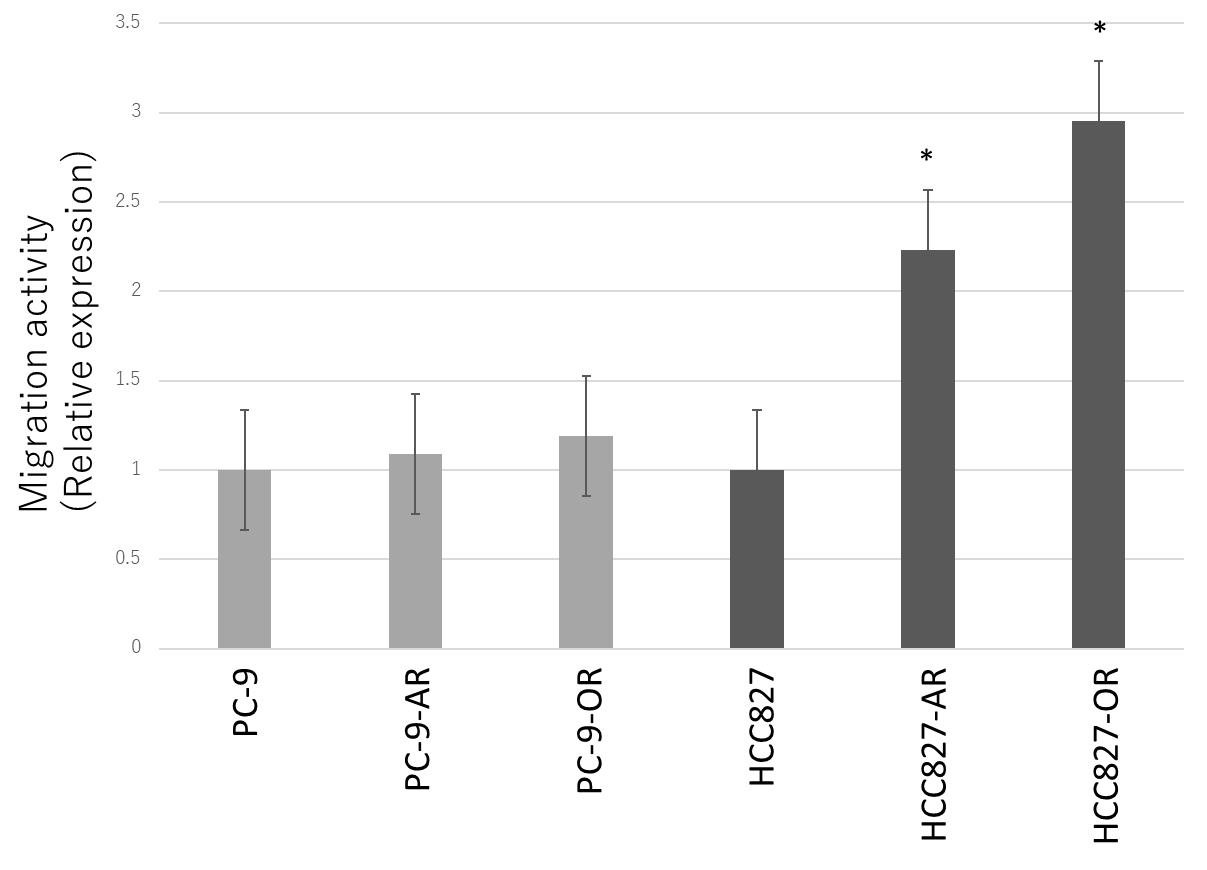
**

**Supplemental Figure S4. RNA interference targeted to ZEB1 resulted in downregulation of ANKRD-1 protein expression.** Relative ZEB1 and ANKRD1 protein expression after ZEB1 silencing by the specific siRNA (si-ZEB1) in A549 and HCC827-OR cells were shown. Relative expression was evaluated using Image Quant (GE Healthcare, IL). NC: negative control siRNA

**
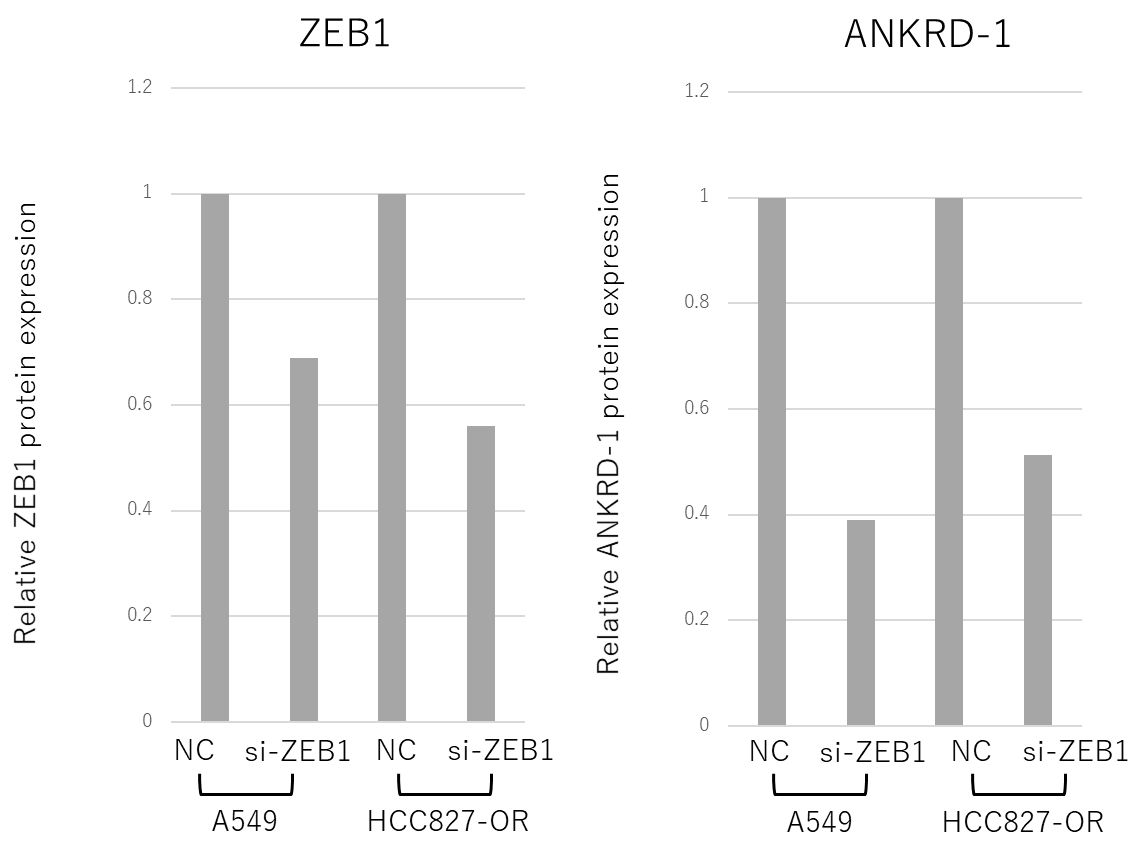
**

**Supplemental Figure S5. Negative and positive staining of ANKRD1 by IHC analysis**

ANKRD1 expression were shown using myocardium tissue as positive control and normal lung as negative control.

**
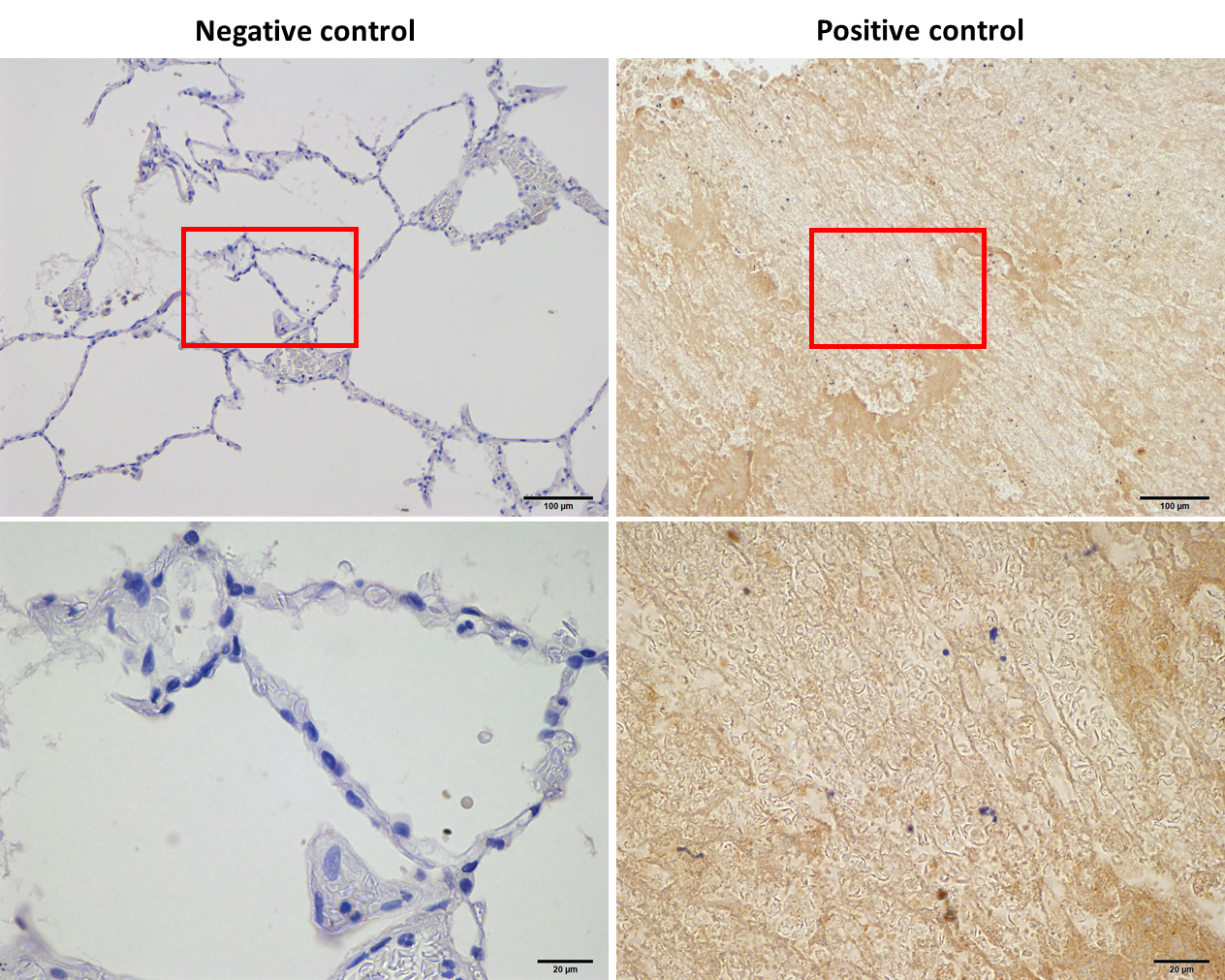
**

**Supplemental Figure S6. E-cadherin expression in EGFR-mutant NSCLC patients before EGFR-TKI therapy by IHC analysis.**

Low E-cadherin expression of patient No. 1 and 2 patients who were treated with EGFR-TKIs for less than 6 months. High E-cadherin expression of patient No. 5 and 7 patients who were treated with EGFR-TKIs for long-term.


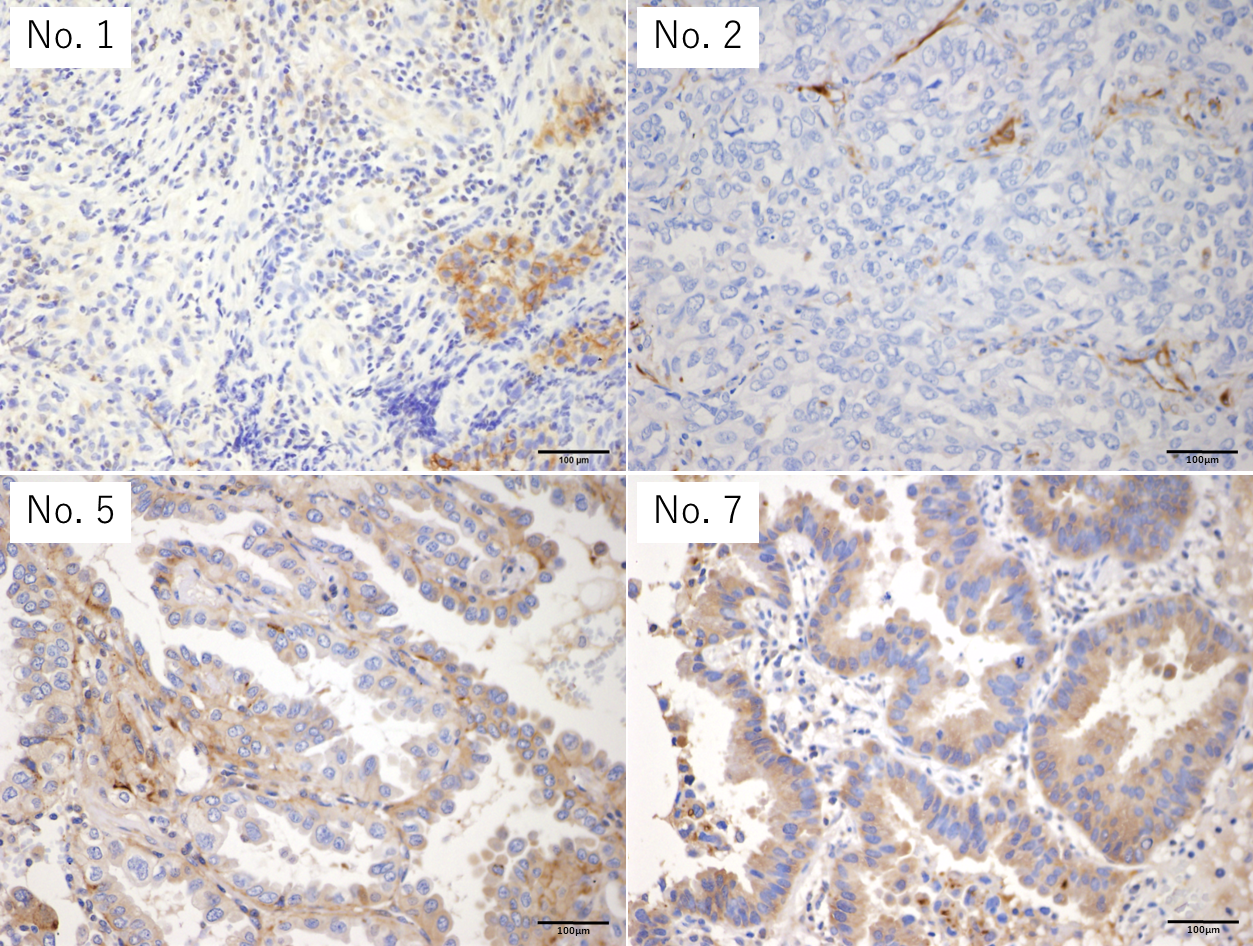


**Supplemental Table 1. E-cadherin and Vimentin expression of lung specimens from EGFR-mutant NSCLC patients before and after EGFR-TKI therapy**

| No. | E-cadherin  Score of Immunostaining | | Vimentin  Score of Immunostaining | |
| --- | --- | --- | --- | --- |
|  | Baseline | After EGFR-TKI | Baseline | After EGFR-TKI |
| 1 | negative | low | negative | negative |
| 2 | negative | high | negative | high |
| 3 | high | negative | negative | low |
| 4 | high | negative | low | high |
| 5 | high | negative | high | high |
| 6 | high | negative | negative | low |
| 7 | high | negative | negative | negative |
| 8 | high | negative | low | low |
| 9 | high | negative | high | high |
| 10 | high | negative | negative | high |

Negative staining (negative); 0~10%, Low grade staining (low); 11~50%; High grade staining (high); 51~100%
